# Antibacterial activity of *Glycyrrhiza glabra *against oral pathogens: an *in vitro *study

**Published:** 2012

**Authors:** Fereshteh Sedighinia, Akbar Safipour Afshar, Saman soleimanpour, Reza zarif, Javad Asili, Kiarash Ghazvini

**Affiliations:** 1 *Department of Biology, Neyshabur Branch, Islamic Azad University, Neyshabur, I. R. Iran *; 2 *Department of Microbiology and virology, Mashhad University of Medical Sciences, Mashhad, I. R. Iran *; 3 *Department of Pharmacology, School of Medicine , Mashhad University of Medical Sciences , Mashhad , I.R. Iran*

**Keywords:** Antibacterial Activity, *Glycyrrhiza glabra*, Oral Pathogen

## Abstract

**Objectives**: Oral infections and dental caries are still considered as serious public health problems and inflict a costly burden to health care services around the world and especially in developing countries.

**Materials and Methods: **In the present study, we evaluated the antibacterial activity of *Glycyrrhiza glabra (G. glabra)* against oral pathogens by diffusion methods and determined the minimum inhibitory concentration (MIC) by both broth and Agar dilution methods and minimum bactericidal concentration (MBC) by broth dilution methods.

**Results: **In this study, *G. glabra* extract showed good antibacterial activity against six bacteria. No strain in this study showed resistance against this extract.

**Conclusion:** *G. glabra*is suggested as an appropriate candidate to help us in order to control dental caries and endodontic infections.

## Introduction

Despite the advances in various field of medicine, oral infections and dental caries are still considered as serious public health problems and inflict a major burden to health care services around the world and especially in developing countries(Singh et al., 2007 ▶; Poole, 2001 ▶). Development of resistance against antibiotics and antiseptics is a growing cause of concern which have limited the preventive measures. Therefore, there is a continuing need to search for new antimicrobial agents (Cai and Wu, 1996 ▶).Over the last decade, plant antimicrobial activity has been studied in different regions of the world and Iran (Janovska et al., 2003 ▶; FazlyBazzaz and Haririzadeh, 2003 ▶). *G. glabra*, commonly called as Licorice, is one of the important traditional medicinal plants grows in the various part of the world and has been used for medicinal purposes for at least 4000 years. Root of this plant has several useful pharmacological properties such as antiinflammatory, antiviral, antimicrobial, and anticancer activities in addition to immunomodulatory, hepatoprotective and cardioprotective effects (Asl and Hosseinzadeh, 2008 ▶). It is a soothing plant that is beneficial in alimentary tract disorders and mouth ulcers (Sanjai, 2005 ▶). Although thereare some studies on antimicrobial activity of Licorice on skin, respiratory, and urinary system pathogens but there is no research about oral pathogens (Ahmad, 2001 ▶). 

In the present study, we evaluated the antibacterial activity of *G. glabra* against oral pathogens.

## Materials and Methods

**Plant material**

*Source, collection and identification*

Roots of *G. glabra* were collected from Garineh, a village near Neyshabour, Iran, during summer 2011. A voucher specimen was prepared and deposited at Research Institute of Plant Sciences Herbarium, Ferdowsi University of Mashhad, Iran.

*Preparation of extract*

Roots of the plant (500gr) were dried at 25˚c and then powdered using a mechanical grinder. The extraction was carried out using ethanol (80%, v/v) for a period of 72 hours without any heating procedure. The final volume of the filtrate was removed using a rotary vacuum evaporator (Heidolphlaborota 4000, Germany) at 40˚c to give the concentrated extract, which was frozen and freeze-dried until use (More et al., 2008 ▶).

**Antibacterial activity**

*Microbial strains*

The microorganisms used in this study included *Streptococcus mutans* (PTCC 1683), *Streptococcus sanguis* (PTCC 1449), *Actinomyces viscosus* (PTCC 1202), *Enterococcus faecalis* (ATCC 29212) as oral pathogens and *Staphylococcus aureus* (ATCC 25923) and *Escherichia coli* (ATCC 29922) as controls. The bacterial strains were cultured in brain heart infusion (BHI) (Difco, MI, USA) under anaerobic condition in an anaerobic jar with Anaerocult A (Merk SA (Pty) Ltd), 37˚c for 72 hours and sub-culturing was done twice weekly. Suspensions of the test organisms were prepared by picking colonies from appropriately incubated agar cultures to sterile broth, to match a McFarland 0.5 turbidity standard (approximately 1.5 x 10^8^ CFU/mL) (McFarland, 1907 ▶).

*Disk diffusion and well diffusion methods*

The microbial growth inhibitory potential of the extract was determined using the agar disk diffusion method as described by CLSI (CLSI, 2009 ▶). The extract was diluted to concentrations ranging from 100 to 3.125 mg/mland chlorhexidine 0.2% mouthwash (ShahrDaru, Tehran, Iran) with concentrations ranging from 0.0625 upto 2 mg/mL and distilled water were used as positive and negative controls, respectively. Twenty microlitre of the plant extract and chlorhexidine concentration were transferred onto sterile filter papers (6.4 mm diameter). Each Mueller-Hinton agar (with 5% sheep blood) was uniformly seeded by means of sterile swab dipped in the suspension and streaked on the agar plate surface. The plates were then incubated at 37◦c for 48 hours anaerobically. All tests were performed in triplicate and zones of inhibition were measured (CLSI, 2009 ▶).

The agar-well diffusion method was performed as prescribed by NCCLSas well. Wells of 5 mm in diameter were punched in the MH agar (with 5% sheep blood) using a sterile cork-borer about 2cm apart. Approximately 20 μl of the extracts were dropped into each well which filled them respectively to fullness. The rest of the process was as mentioned previously (NCCLS, 2000 ▶).

**Determination of minimum inhibitory concentration (MIC) and minimum bactericidal concentration (MBC)**

*Macro broth dilution method*

The minimum inhibitory concentration (MIC) of the extracts was determined according to methods described by CLSI 2006. *G. glabra* extract was diluted to concentrations ranging from 100 to 0.78 mg/mL in Mueller Hinton broth. To each dilution tubes, 0.1 ml of the bacterial inoculumwas seeded. Control tubes with no bacterial inoculation were simultaneously maintained. Tubes were incubated anaerobically at 37°c for 24 hours. The lowest concentration of the extract that produced no visible bacterial growth (turbidity) was recorded as the MIC (CLSI, 2006 ▶). To estimate the MIC of the extract more precisely and for confirmation of the results, a more precise concentration in agar dilution method was used.

*Agar dilution method*

Agar dilution assay was used to test the susceptibility of the microorganisms to the *G. glabra *extract at different concentrations, as recommended by the Clinical Laboratory Standards Institute (CLSI).Serial dilutions of the *G. glabra* extract were prepared in plates according to the standard procedure. After solidification, the plates were incubated at 37°c for 2 hours in order to dry the agar surface. The assay plates were estimated to have 50, 35, 30, 25, 20, 12.5, 10, 6.25, 3.125, 2.5 and 1.25 mg/ml of active Licorice extract. Inocula were applied to agar surfaces in 1 L spots, giving approximately 1.5 x 10^5^cfu per spot. Plates without added extract were inoculated as viability controls and uninoculated media were also included to confirm sterility. All plates were inverted and incubated appropriately for 48 to 72 hours in Gas Pak jars. The MIC was considered as the lowest concentration of extract which caused a marked inhibition in growth as compared to the growth control. This extract was tested in triplicate vs. each organism (three separate inoculums preparations on three different days)(CLSI, 2009 ▶).

## Results

In vitro antibacterial activity of *G. glabra* and their potency were quantitatively and qualitatively assessed by determining the inhibition zone diameter and MIC as given in Tables 1-4. Screening results of antibacterial activity of this plant against six bacteria are shown in Tables 1 and 2.

The analysis of *G. glabra* extract showed positive inhibitory activity against six bacteria, in all methods. No strain in this study showed resistance to this extract. The inhibitory zone significantly increased in a dose dependent manner. 

In agar dilution method Minimum inhibitory concentration (MIC) for *Streptococcus mutans*, *Actinomyces viscosus* and *Enterococcus faecalis* were 12.5 mg/ml and for *Escherishia coli* and *Staphylococcus aureus* were 35 mg/ml. MIC for *Streptococcus sanguis*was 30 mg/ml. *E. coli*demonstrated the greatest resistance to *G. glabra* and appeared to be the most resistant bacterium (Table 3). For these microorganisms, MIC of chlorhexidine mouthwash was 0.0625 mg/ml except for *E.coli* that was 0.125 mg/ml (Table 7 and 8) .The results of broth dilution are shown in table 4 which are consistent with the findings of the agar dilution.

**Table 1:** Antimicrobial activity of the plant tested against oral microorganisms and controls with zones of inhibition in millimetre of the extractin disk diffusion method.

**Plant extract**	**Concentration**	***S. mutans***	***S. sanguis***	***A. viscosus***	***E. faecalis***	***S. aureus***	***E. coli***
***Glycyrrhizaglabra***	100 mg/ml	26.5±0.8	14.4±0.4	25.4±0.3	24±0.0	25.1±0.2	22.5±0.2
	50 mg/ml	23±0.3	12.2±0.4	22.4±0.4	20.3±0.1	22.4±0.5	20±0.0
	25 mg/ml	18.3±0.4	10±0.0	17.6±0.5	15±0.0	18±0.0	16.2±0.2
	12.5 mg/ml	17.1±0.2	7.6±0.7	16.2±0.2	9.2±0.2	14.4±0.4	13±0.0
	6.25 mg/ml	12.9±0.5	-	12±0.0	6±0.0	9.2±0.2	9±0.0
	3.125 mg/ml	9.3±0.4	-	8.3±0.2	-	-	-
**Negative control**		­	­	­	-	-	-

- : No inhibition zone

These results showed that antibacterial activity of this extract was significantly greater than negative control (p value less than 0.05).

**Table 2:** Antimicrobial activity of the plant tested against oral microorganisms and controls with zones of inhibition in millimetre of the extract in well diffusion method.

**Plant extract**	**Concentration**	***S.mutans***	***S.sanguis***	***A.viscosus***	***E.faecalis***	***S.aureus***	***E.coli***
***Glycyrrhizaglabra***	100 mg/ml	27.3±1.3	16.4±0.4	26.1±0.2	23.8±0.8	24.6±0.5	23.8±0.7
	50 mg/ml	23.1±0.6	12±0.0	20.7±0.4	16.4±0.5	20.6±0.5	19.1±0.8
	25 mg/ml	20±0.0	10±0.0	18.1±1	16±1	17±0.0	15±0.0
	12.5 mg/ml	17.6±1	8.2±0.2	16.1±0.2	11±0.0	12.6±0.6	12.4±0.5
	6.25 mg/ml	15.7±0.9	-	13.1±0.2	8±0.0	9.1±0.7	9±1.4
	3.125 mg/ml	12.1±0.2	-	11±0.0	-	-	-
**Negative control**		-	-	-	-	-	-

- : No inhibition zone

Theresults obtained by above mentioned method confirmed that antibacterial activity of this extract was significantly greater than negative control (p value less than 0.05).

**Table 3:** Mean MIC (mg/ml) results of *Glycyrrhiza* *glabra* extract on oral microorganisms and controls in agar dilution method.

	***S. mutans***	***S. sanguis***	***A. viscosus***	***E. faecalis***	***S. aureus***	***E. coli***
**MIC**	12.5	30	12.5	12.5	35	35

**Table 4:** Mean MIC and MBC (mg/ml) results of *Glycyrrhiza* *glabra* extract on oral microorganisms and controls in broth dilution method

	***S. mutans***	***S. sanguis***	***A. viscosus***	***E. faecalis***	***S. aureus***	***E. coli***
**MIC**	12.5	50	12.5	12.5	50	50
**MBC**	12.5	50	12.5	12.5	50	50

**Table 5:** Antimicrobial activity of the chlorhexidin against oral microorganisms and controls with zones of inhibition in millimetre of the extract in disk diffusion method.

	**Concentration**	***S. mutans***	***S. sanguis***	***A. viscosus***	***E. faecalis***	***S. aureus***	***E. coli***
**Chlorhexidin**	2 mg/ml	26.2±0.2	17.2±0.2	23±0.0	25.2±0.2	26±1	24±0.2
	1 mg/ml	22.2±0.2	16±0.0	17.7±0.4	22.2±0.1	23±0.5	21.2±0.0
	0.5 mg/ml	18±0.0	15.2±0.2	13.7±1	17.4±0.7	19±0.0	18.4±0.99
	0.25 mg/ml	14±0.0	11.7±0.99	11.7±0.4	11±0.0	15.4±0.7	15±0.0
	0.125 mg/ml	10±0.0	10±0.0	9.5±0.7	8.2±0.2	11.2±0.2	12±0.0
	0.625 mg/ml	8.5±0.7	-	7.2±0.2	6±0.0	8±1	-
**Negative control**		-	-	-	-	-	-

- : No inhibition zone

These results showed that antibacterial activity of chlorhexidinas a well-known antibacterial agent was not significantly greater than *Glycyrrhiza glabra* extract (p value more than 0.05).

**Table 6:** Antimicrobial activity of the chlorhexidin against oral microorganisms and controls with zones of inhibition in millimetre of the extract in well diffusion method

**Plant extract**	**Concentration**	***S. mutans***	***S. sanguis***	***A. viscosus***	***E. faecalis***	***S. aureus***	***E. coli***
**Chlorhexidin**	2 mg/ml	28.2±0.2	20±0.0	22.2±0.2	±0.0	±0.2	±0.2
	1 mg/ml	23.9±0.7	16.7±0.4	18.2±0.2	±0.1	±0.5	±0.0
	0.5 mg/ml	20.2±0.2	14.2±0.2	14.7±0.4	±0.0	±0.0	±0.2
	0.25 mg/ml	14.4±0.0	11.2±0.2	12.5±0.1	±0.2	±0.4	±0.0
	0.125 mg/ml	12±0.0	9.7±0.4	10.2±0.2	±0.0	±0.2	±0.0
	0.625 mg/ml	9.4±0.5	-	8±0.0	-	-	-
**Negative control**		-	-	-	-	-	-

- : No inhibition zone

Theresults obtained by above mentioned method confirmed that antibacterial activity of chlorhexidinas a well-known antibacterial agent was not significantly greater than *Glycyrrhiza glabra* extract (p value more than 0.05).

**Table 7:** Mean MIC (mg/ml) results of chlorhexidinextract on oral microorganisms and controls in agar dilution method

	***S. mutans***	***S. sanguis***	***A. viscosus***	***E. faecalis***	***S. aureus***	***E. coli***
**MIC**	0.0625	0.0625	0.0625	0.0625	0.0625	0.125

**Table 8:** Mean MIC and MBC (mg/ml) results of chlorhexidin extract on oral microorganisms and controls in broth dilution method

	***S. mutans***	***S. sanguis***	***A. viscosus***	***E. faecalis***	***S. aureus***	***E. coli***
**MIC**	0.0625	0.0625	0.0625	0.0625	0.0625	0.125
**MBC**	0.125	0.125	0.0625	0.125	0.125	0.125

## Discussion

The antimicrobial activity of root extract of *G. glabra* has been shown in some other studies but antibacterial effects of this plant against oral pathogens has not been studied (Shapna et al., 2010 ▶; Meghashri et al., 2009 ▶). The present study supports the view that *G. glabra* root extract might be useful as antibacterial agents against oral pathogens. The findings of this study propose that *G. glabra* can inhibit the growth of *Streptococcus mutans, Actinomyces viscosus, Streptococcus sanguis, *and* Enterococcus faecalis.*

The ethanolic extract of *G. glabra* had promising MIC value against all oral bacteria especially *S. mutans, A. viscosus, and E. faecalis*. Although in some studies, it has been reported that *G. glabra* extract has antibacterial activity against several bacteria such as *S. aureus, E. faecalis, *and *E. coli,* but there are a few studies about oral pathogens such as *A. viscosus and S. sanguis *(Nirmala et al., 2011 ▶). In this report, antibacterial activity of this plant against *A. viscosus* evaluated for the first time. 

In the present study, this plant showed antibacterial activity against *S. aureus *but it is interesting that *G. glabra* root extract did not show any antimicrobial activity when tested against this microorganism in another study (Nirmala et al., 2011 ▶).In one study it was shown that Glabridine,one of the most important substances in this plant, had antibacterial activities against some strains and it was more active against gram positive strains than gram negative (Vivek *et al.*, 2008). In the present study, ethanolic extract of this plant exhibited the highest MIC value against *E. coli*, so maybe antibacterial activity of *G. glabra* against gram positive bacteria was more than gram negative bacteria. 

The prevalence of dental caries, as one of the major problems in oral health, has caused increased use of mouthwash products. Herbal mouthwashes, compared with chemical drugs, have fewer side effects and are more economical. This in-vitro study suggests *G. glabra* as a candidate which can help us to control dental caries and endodontic infections. The effects of this extract maybe morebeneficial if it is incorporated in gum, toothpaste, mouthwash, and dental products to reduce plaque and dental caries.

Further studies are required to better evaluate the effect of this extract if used as endodontic irrigants and *In vivo* clinical testing is essential to confirm the*in vitro* results.
